# The clock is ticking on schizophrenia: a study protocol for a translational study integrating phenotypic, genomic, microbiome and biomolecular data to overcome disability

**DOI:** 10.3389/fpsyt.2024.1451678

**Published:** 2024-10-30

**Authors:** Giacomo Mercuriali, Lorenzo Lodde, Pasquale Paribello, Jacopo Sapienza, Alice Corona, Chiara Ave, Delia Pacini, Daniela Nocera, Carolina Corrias, Sabrina El Kacemi, Michele D'Incalci, Ilaria Frau, Elena Monzani, Flavia Valtorta, Donatella Congiu, Anna Meloni, Maria Scherma, Paola Fadda, Simona Dedoni, Carlotta Siddi, Stefania Sut, Stefano Dall’Acqua, Sofia Nasini, Benedetta Barzon, Alessio Squassina, Roberto Cavallaro, Mirko Manchia, Claudia Pisanu, Marta Bosia, Stefano Comai

**Affiliations:** ^1^ IRCCS San Raffaele Scientific Institute, Milan, Italy; ^2^ Unit of Psychiatry, Department of Medical Sciences and Public Health, University of Cagliari, Cagliari, Italy; ^3^ Unit of Clinical Psychiatry, University Hospital Agency of Cagliari, Cagliari, Italy; ^4^ School of Medicine, Vita-Salute San Raffaele University, Milan, Italy; ^5^ Department of Biomedical Science, Section of Neuroscience and Clinical Pharmacology, University of Cagliari, Monserrato, Cagliari, Italy; ^6^ Department of Pharmaceutical and Pharmacological Sciences, University of Padua, Padua, Italy; ^7^ Department of Pharmacology, Dalhousie University, Halifax, NS, Canada; ^8^ Department of Biomedical Sciences, University of Padua, Padua, Italy; ^9^ Department of Psychiatry, McGill University, Montreal, QC, Canada

**Keywords:** schizophrenia, cognitive function, metabolic syndrome, tryptophan, kynurenine, melatonin system, microbiota

## Abstract

**Background:**

Shared biological factors may play a role in both the cognitive deficits and the increased prevalence of metabolic syndrome observed in individuals with Schizophrenia (SCZ). These factors could entail disturbances in tryptophan (Trp) to both melatonin (MLT) and kynurenine (Kyn) metabolic pathways, as well as inflammation and alterations in the gut microbiome composition.

**Methods:**

The present research project aims to investigate this hypothesis by recruiting 170 SCZ patients from two different recruitment sites, assessing their cognitive functions and screening for the presence of metabolic syndrome. Additionally, we plan to assess the impact of a 3-month cognitive remediation therapy on 30 of these patients. We will analyze clinical data alongside serum biomarkers and gene expression related to the Trp- to MLT and Kyn metabolic pathways, markers of inflammatory and composition of the gut microbiome. The association between Trp-MLT-Kyn levels, expression levels of selected genes, inflammatory markers and clinical phenotypes will be analyses in the context of general linear models.

**Discussion:**

This project has the potential to identify some typical SCZ symptomatic clusters that will be more stringently associated with variations in the Trp-MLT-Kyn/inflammatory system and with a better response to cognitive remediation therapy. Moreover, in a future perspective, it may highlight a group of patients who may benefit from a pharmacological treatment aiming at reinstating the physiological Trp to MLT and Kyn system. Therefore, it has the potential to move research toward a personalized approach for SCZ management.

## Background

Schizophrenia (SCZ) is a chronic and highly complex psychiatric disorder characterized by significant disability. It has a median point prevalence of 4.6 cases per 1000 individuals (1). Despite the relative rarity of SCZ and the discrete inconsistencies surrounding estimates of disease burden, the negative impact on patients’ functionality and quality of life is increasingly evident (2, 3). The treatment of schizophrenia continues to present several significant challenges, primarily drug resistance and cognitive impairment, the latter affecting over 80% of patients and persisting even after a good antipsychotic response (4, 5).

Additionally, metabolic syndrome (MetS), which affects over 30% of patients with SCZ, can be aggravated by antipsychotic therapies and, in turn, worsens the overall clinical condition (6, 7). Pharmacological treatments for cognitive and metabolic disturbances in SCZ remain unsatisfactory because of their limited efficacy and tolerability concerns. At the same time, non-pharmacological interventions, such as Cognitive Remediation Therapy (CRT), show heterogeneous results. These clinical unmet needs reflect the gap in knowledge about their underlying biological and molecular mechanisms.

Alterations of the circadian rhythms, regulated mainly by the suprachiasmatic nucleus in the hypothalamus, may represent a common biological denominator for both cognitive and metabolic dysfunctions in SCZ (8). For example, the circadian rhythmicity of genes involved in mitochondrial function and inflammatory response is significantly disrupted in the prefrontal cortex of SCZ patients (9). One of the major regulators of the activity of the suprachiasmatic nucleus is the neuromodulator melatonin, which primarily acts through two G-protein coupled receptors, MT1 and MT2. These two receptors are expressed not only in the suprachiasmatic nucleus but also in many other regions of the brain (10). MLT is synthesized from tryptophan (Trp) along the serotonin (5-HT) pathway by the enzyme tryptophan hydroxylase. Immune activation and inflammation can reduce the availability in the brain of Trp for the synthesis of 5-HT and MLT because they induce an enhanced degradation of the amino acid along the kynurenine (Kyn) pathway, with the formation of downstream neuroactive compounds, among which quinolinic acid (QUIN) and kynurenic acid (KYNA) (11).

More specifically, in the brain KYNA is synthesized by astrocytes and acts as an antagonist on ionotropic glutamate receptors, exhibiting the highest affinity for N-methyl-D-aspartate (NMDA) receptors. Moreover, it has shown inhibitory activity of α7 nicotinic acetylcholine (α7nACh) receptors and thus may be important in suppressing presynaptic glutamate release (12, 13).

Both functions confer neuroprotective properties to KYNA, as it reduces excitotoxicity, a pathological process implicated in various brain disorders, by decreasing presynaptic glutamate release and preventing the hyperactivation of NMDA receptors. These activities balance the neurotoxic properties of QUIN that acts as NMDA agonist at the glutamate site. The neuroprotective properties of KYNA are relevant at physiological concentrations of the metabolite. In contrast, an abnormal and excessive increase of KYNA leads to NMDA hypofunction, a condition associated with the pathogenesis of positive and negative symptoms but also with the cognitive symptomatology of SCZ. Indeed, KYNA levels seem to be higher in individuals with SCZ and their unaffected siblings, but also to be associated with the onset of psychotic symptoms and cognitive deficits (14–17). Additionally, KYNA levels negatively correlate with Cortex Grey Matter Volume in SCZ (18). Importantly, KYNA levels tend to decrease or return to normal after remission (19).

As mentioned above, QUIN is the counterpart of KYNA. QUIN activates NMDA receptors, leading to excitotoxicity as the result of an excessive influx of calcium ions into neurons. This excessive Ca^2+^ influx triggers various downstream processes, including the generation of reactive oxygen species, mitochondrial dysfunction, activation of nitric oxide synthase (NOS) and ultimately, neuronal cell death (20, 21).

KYNA and QUIN produced peripherally cannot reach the brain because they do not cross the brain-blood-barrier (BBB) due to their polarity. Therefore, only KYNA and QUIN produced directly within the CNS, by astrocytes and microglial cells, respectively, likely play a role in the pathogenesis of SCZ symptoms and comorbidities.

However, the accumulation of adipose tissue is known to contribute to the worsening of MetS due to the massive secretion of inflammatory cytokines, which, in addition to exerting systemic effects on metabolism, are able to increase the permeability of the BBB. Consequently, a potential crossing of the BBB by KYNA and QUIN in SCZ associated with severe MetS cannot be completely excluded.

If this was true, considering that 60% of brain KYN comes from the periphery, it is possible that peripheral inflammation increases central KYN concentrations. Furthermore, dysregulation of TRP metabolism along the KYN pathway is one of the mechanisms underlying insulin resistance and metabolic abnormalities in the general population (22). Interestingly, circulating levels of QUIN seem to correlate with MetS in SCZ (23).

The activation of the Kyn pathway also appears to be associated with increased cognitive deficits in patients with SCZ. In particular, inhibition of NMDARs and α7nAChRs reduces the levels of glutamate, dopamine, and acetylcholine, which may be associated with altered cognitive performances (24–27). In addition, Trp depletion as a consequence of the increased activation of the KYN pathway may also play a role, leading to a reduced amount of the amino acid for the synthesis of serotonin (5HT).

Considering all these potential factors implicated in the pathophysiology of SCZ and comorbidities, the Kyn pathway may be at the intersection of neuroinflammation and neurotransmission since inflammatory cytokines by upregulating specific enzymes such as indoleamine-2,3-dioxygenase, tryptophan-2,3-dioxygenase, and kynurenine 3-monooxygenase, can elevate the amount of Trp metabolized by this pathway, causing an imbalance between neurotoxic and neuroprotective metabolites on one side, and reduced synthesis of 5-HT on the other side. It is well known that pro-inflammatory cytokines like IFN-γ, IL-6 and oxidative stress markers (ROS) increase the catabolism of Trp into KYN. Interestingly, our previous report described a greater activation of the Kyn pathway in individuals with treatment-resistant SCZ compared to those responding to the first line of treatment, suggesting that a greater activation of the pathway might impact treatment response (28).

A key aspect of SCZ psychopharmacology is the fact that current antipsychotic medication, which represent the cornerstone of pharmacological treatment for psychotic disorders, show minimal effects on cognitive impairments (29). CRT is a behavioral intervention that involves intensive task practice and learning strategies to create lasting improvements in cognitive skills (30). Increasing evidence shows small to moderate improvements in cognitive and functional outcomes for CRT in SCZ (31). CRT protocols vary in the cognitive domains that they target (e.g., attention, memory), the sensory modalities they utilize (e.g., visual, auditory, multi-sensory), and their delivery format (e.g., computerized, group-based, therapist-led). Furthermore, CRT protocols differ in their employed strategies, with some approaches focusing more on lower-level cognitive processes as opposed to protocols focusing more on higher cognitive functions (such as problem-solving). Over the past several decades, a growing body of research has assessed the efficacy of CRT in addressing various cognitive dysfunctions in patients with SCZ (32). The association between sleep disturbances, MetS and cognitive dysfunction has been described in both clinical populations (33, 34) and in non-clinical populations (35) with the existing evidence pointing at a sizeable impact of MetS on cognitive performance. Considering the frequency of both sleep disorders and MetS in individuals living with SCZ, this association appears particularly relevant in the clinical management, especially in light of the potential negative impact of numerous psychotropic medications on metabolic parameters (36).

It is important to mention that a frequent critical factor inducing inflammation in SCZ is MetS, which is significantly associated with neuroinflammation and alteration of the BBB (37). As mentioned earlier, neuroinflammation is associated with increased activation of the KYN pathway (38, 39), which further appears to impact cognitive abilities (40).

Various studies have explored the relationship between MetS and cognitive performance in schizophrenia. Although some conflicting findings suggest paradoxically better cognitive performance in patients with schizophrenia and hypertension (41), there is a general consensus that MetS and its components contribute to the cognitive decline typically observed in schizophrenia (42, 43). Moreover, evidence indicates that MetS in schizophrenia may also influence the degree of cognitive improvement achievable through cognitive remediation. In particular, a significantly negative effect of MetS was observed on executive functions, typically impaired in schizophrenia, as well as on global cognitive efficiency (44). MetS may impair cognitive performance and limit improvements by promoting neuroinflammation, leading to alterations in brain microcirculation and gray matter microstructure (45).

Consistent links have been established between long-term disruption in sleep, circadian machinery, inflammation and increased risk of developing MetS and cognitive impairments (46–48) it is plausible that dysregulation of the KYN pathway may represent a common neurobiological marker for both cognitive and metabolic dysfunction in SCZ.

Interestingly, the MLT system also influences insulin secretion, lipid and glucose homeostasis (49, 50), with the MLT system frequently appearing dysfunctional in SCZ patients in terms of MLT levels, synthesis and secretion (51). Finally, pathways involving Trp, MLT, Kyn and inflammation are strongly linked to the gut microbiome, which in turn also influences brain function (52). Indeed, administering MLT in mice subjected to a high-fat diet (HFD) results in decreased body weight and lower-grade systemic inflammation, improved insulin resistance, and significant change in intestinal microbiome composition (53). Recent yet limited evidence shows alterations in gut microbiome composition that are specific to SCZ and correlated with symptom severity (54). Altogether, these findings lead us to hypothesize a relationship between the Trp/5-HT/MLT/Kyn and the inflammatory systems, the microbiome, MetS and cognitive function in SCZ, potentially allowing the identification of new biomarkers and treatment targets. Indeed, a comprehensive investigation that integrates preclinical and clinical studies exploring the neurobiological, genomic and microbiome changes within the Trp to MLT-circadian and Kyn systems and inflammation linked with MetS and cognition in SCZ is currently lacking.

The role of the kynurenine pathway in schizophrenia is further supported by preliminary evidence from our research group. Unpublished data suggest a greater effect of the kynurenine pathway in patients with more pronounced cognitive deficits; also showing a predictive effect of QUIN/KYNA, 3-HK/KYN and TNF-α on the magnitude of cognitive improvement post CRT, underscoring the potential role in dynamic modulation of cognition (55). Moreover, in patients with SCZ, cognitive resources (Intelligent Quotient, IQ), as measured by the Wechsler Adult Intelligence Scale (WAIS-R), appear to positively correlate with plasma Trp levels (unpublished data).

The goal of this project is thus to identify distinct biological markers associated with MetS and cognitive function in SCZ through a combination of clinical and preclinical analyses and to assess how the modulatory impact of CRT influences these identified risk markers. These findings will enhance our understanding of SCZ and provide new insights for tailoring treatment approaches to individual patients.

## Objectives of the study

To our knowledge, this is the first study to address the common neurobiological mechanisms underlying two of the main unmet needs in the treatment of SCZ, i.e. metabolic and cognitive dysfunctions, which are some of the main determinants of disability in this condition.

Our primary objective in the clinical arm of the project is to test the association between TRP/5-HT/MLT and KYN pathways, inflammation, gene expression levels, composition of the gut microbiome and clinical phenotypes in patients with SCZ in order to identify distinct biological patterns (biosignatures) that discriminate patients according to the severity of MetS and cognitive dysfunction.

Secondary objectives include 1) identifying phenotypic and neurobiological determinants associated with response to CRT after 36 sessions (one session three times per week, for a total of 12 weeks); 2) testing the association between TRP/5-HT/MLT and KYN biomarkers, inflammation, gene expression levels and gut microbiome composition with quality-of-life levels in SCZ patients; 3) evaluating the association between sleep quality, severity of metabolic syndrome, cognitive dysfunction and their interaction with changes in the Trp-5-HT-MLT-Kyn/inflammatory system.

In a personalized medicine perspective of SCZ, this project has the potential to identify certain symptomatic clusters typical of SCZ that will be closely associated with changes in the Trp/5-HT/MLT/Kyn/inflammatory system with a better response to CRT.

### Study population

Our population of interest includes patients with SCZ who are followed as outpatients or who are currently hospitalized in one of the two centers involved in the study.

### Inclusion criteria

The inclusion criteria for our study are (1) age 16-65 years; (2) diagnosis of schizophrenia according to the diagnostic criteria of the Diagnostic and Statistical Manual of Mental Disorders, Edition 5^th^ (DSM-5; American Psychiatric Association, 2014); (3) being treated with antipsychotic medication; and (4) being able to give written informed consent.

### Exclusion criteria

The exclusion criteria are (1) diagnosis of dementia; (2) comorbidity with other active psychiatric disorders; (3) severe medical/neurological conditions in the absence of adequate clinical compensation and (4) patients with known chronic inflammatory diseases as well as patients in a clinically detectable acute inflammatory state were excluded from the study.

### Study design and recruitment process

**Figure 1:**
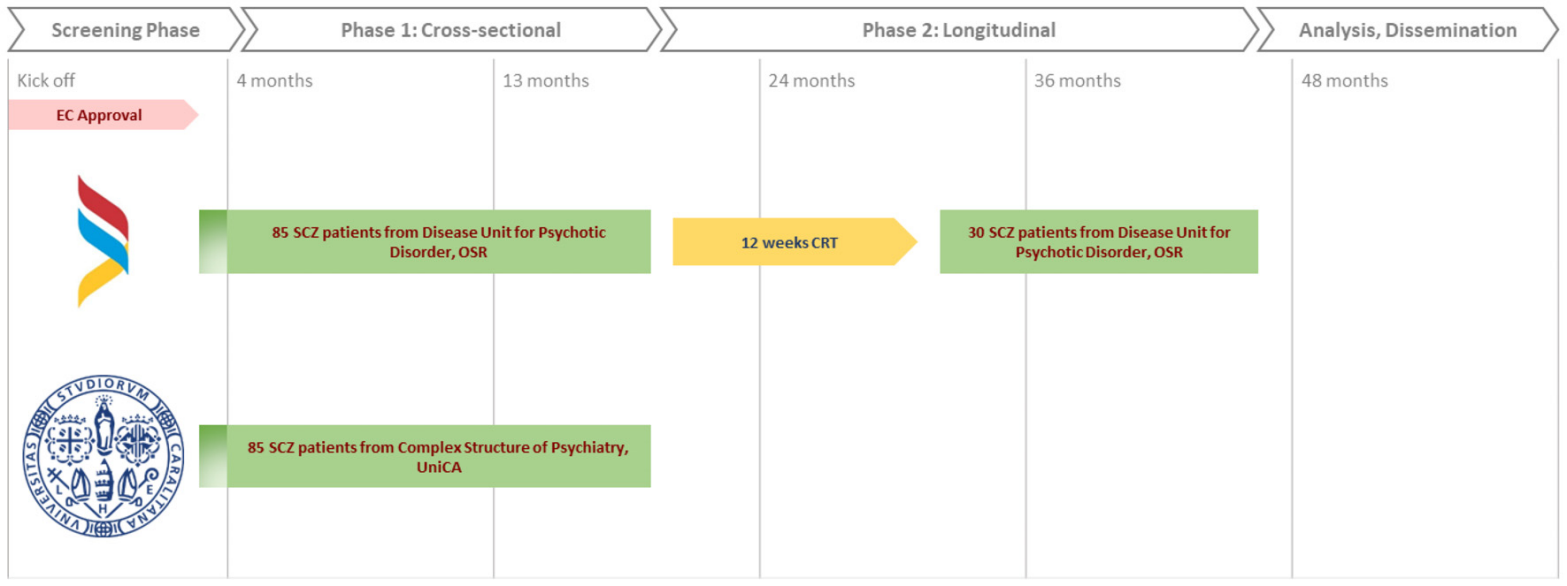
Timeline of SHERRIE Trial.

The study is articulated in two phases, as depicted in **Figure 1**. The first phase is a cross-sectional study aiming at probing the association between clinical variables (metabolic syndrome, cognitive dysfunction, quality of life and sleep quality) and biological variables (TRP/5-HT/MLT, KYN, inflammation, gene expression levels and microbiome) in the total sample of 170 patients. We plan to recruit 85 patients with schizophrenia from each of the two centers: Complex Structure of Psychiatry of the University of Cagliari, Italy, and the Psychotic Disorders Unit of the San Raffaele Hospital, Milan, Italy.

The second phase will involve a prospective longitudinal cohort evaluation of the same clinical and biological variables in relation to the response to CRT (Cognitive Remediation Therapy) after 12 weeks of treatment. This study phase will involve a subsample of 30 subjects randomly drawn from the pool of 85 patients enrolled in phase 1 at the Psychotic Disorders Unit of the San Raffaele Hospital, Milan. Italy.

Recruitment of patients deemed suitable for the study will take at least 36 months.

### Sample size estimation

Previous estimates of effect sizes for variation of peripheral MLT (51), gene expression (52) and gut microbiome composition (36) in schizophrenia show that 170 patients are sufficient to achieve 90% statistical power to detect a correlation between the selected peripheral markers, genomic signatures and disease profiles, with an alpha=0.05. Considering the longitudinal pilot study, given its nature and the highly adaptable nature of the selected cognitive rehabilitation regimen, we expect an improvement in the studied clinical outcomes for a wide range of patients recruited in phase two. However, considering the study’s exploratory nature in both phases of the study and a lack of a formally operationalized cognitive deficit threshold as an inclusion criterion for the interventional phase, at this stage, no effect size estimation is possible for either the impact of the intervention on clinical outcomes or for its association with the selected translational biomarkers.

### Specific objective and experimental design, clinical assessment

#### Phase 1

To investigate the associations between TRP/5-HT/MLT, KYN, inflammation, gene expression levels, gut microbiome composition, and clinical phenotypes in patients with schizophrenia (Primary Aim), and to stratify patients based on the severity of MetS and cognitive dysfunction, as well as their associations with sleep quality and specific clinical phenotypes, we designed Phase 1 of the study. During the screening phase and phase 1 (cross-sectional study), all patients will undergo a formal clinical assessment comprising anthropometric measurements (weight, height, BMI, waist circumference, hip circumference, blood pressure and heart rate) and blood tests frequently employed to diagnose MetS according to the criteria of the International Diabetes Federation (2005, i.e., total cholesterol, high-density lipoproteins, low-density lipoproteins, fasting blood glucose, Insulin, glycated hemoglobin, C-Reactive Proten, aspartate aminotransferase, alanine aminotransferase, gamma-glutamyl transferase, azotemia, creatinine and triglycerides).

The following will be assessed:

- *primary clinical outcomes* with Positive and Negative Syndrome Scale (PANSS), Brief Assessment Cognition Schizophrenia (BACS, the presence and degree of cognitive deficits are based on the equivalent scores of the Italian version) (56); *secondary outcomes* with the Quality-of-Life Scale (QLS), and the Pittsburgh Sleep Quality Index (PSQI (57).
- *biological biomarker candidates* in fasting blood sampling and measurement of Trp/5-HT/MLT and Kyn pathways metabolites in plasma (Trp, 5-HT, MLT, Kyn, KYNA, QUIN); inflammatory markers (IL-1B, Interleukin 6 (IL-6), Tumor Necrosis Factor Alpha (TNF-a), high-sensitivity C-reactive protein (CRP)); a stool sample for gut microbiome analysis through 16S rRNA sequencing; gene expression analysis by RNA sequencing to measure the expression of selected genes related to Trp/5-HT/MLT and Kyn pathways and inflammation: MTNR1A; MTNR1B; tryptophan hydroxylase 1 and 2; aralkylamine N-acetyltransferase; indolamine 2,3-deoxygenase 1 and 2 (IDO1 and IDO2); kynurenine 3-monooxygenase (KMO); monoamine oxidase A (MAO-A); IL-1B, IL-6, TNF-a and CRP.

#### Phase 2

To identify the phenotypic and neurobiological determinants of the response to CRT, phase 2 (longitudinal pilot study) was designed to examine whether the response to CRT is associated with changes in the biosignature described for the primary objective in addition to a baseline clinical assessment.

For phase 2, 30 patients will be randomly selected from the San Raffaele Hospital group to be treated with CRT for a duration of 3 months (12 weeks). At the end of CRT, the patients will be re-evaluated for the same clinical and biological variables, and with the same methods, analyzed during phase 1.

The selected CRT protocol, initially developed for brain-injured patients, is a neuropsychological deficit treatment modality based on the concept that by intensively exercising inefficient functions in the patient, these can be enhanced. It targets specific deficits and aims at a generalized improvement in functioning. It consists of two weekly one-hour sessions of specific computerized exercises for a period of 3 months (12 weeks), for a total of 36 hours. The computer-assisted neurocognitive exercises were performed using Cogpack software (58). Exercises will be selected specifically for each patient based on his or her performance at baseline, and then the program will set up for adaptive exercises based on the subject’s performance during the session; there are initial trial sessions for each exercise to promote confidence in using the software. A domain-specific exercise will be included for each poor performance (equivalent score 0-1), and for each good performance (equivalent score 2-4), a non-domain-specific exercise will be added. The exercises will be administered by trained psychologists (59). The exercises have visual and auditory performance feedback. For each “bad” performance, the assigned exercise will correspond to the most deficient cognitive domain (executive functions, sustained attention, verbal memory, working memory, motor activity/coordination, verbal fluency, and selective attention). We expect that the possible improvements in cognitive functions will reflect on other clinically relevant areas, such as the quality of life; one of the purposes of this study is to understand the possibly associated biological correlates for the observed clinical improvements.

### Collection and analysis of biological samples

The collection of biological samples consists of the following:

**-** Peripheral venous blood sampling aimed at evaluating the plasma levels of Trp, 5-HT, MLT, KYN, QUIN, KYNA, inflammatory factors (interleukin 1 and 6, tumor necrosis factor-alpha and C-reactive protein) and related analysis of their gene expression.

**-** Stool sample collection for the evaluation of the intestinal microbiome composition.

Blood samples intended to measure metabolites of the Trp metabolism and circulating inflammatory factors will be sent to the Neuropsychopharmacology Laboratory of the San Raffaele Institute.

The stool samples collected, together with the blood samples for gene expression analysis, will be sent to the Pharmacogenomics Laboratory of the Department of Biomedical Sciences of the University of Cagliari for RNA extraction, library preparation and sequencing at the Centre for Research University Services (CeSAR) of the University of Cagliari. The samples will be stored for five years and subsequently destroyed.

Plasma levels of IL-6, IL-1B, TNF-a and CRP will be quantified with highly sensitive sandwich-ELISA kits. Gene expression analysis will be performed with RNA sequencing on an Illumina NextSeq 2000 platform. Quality control, pre-processing and alignment with the reference genome will be performed with the rnaseq nf-core bioinformatic pipeline (60). Microbial DNA will be isolated from stool samples and used for amplification and sequencing of the 16S rRNA gene pool on an Illumina MiSeq system. Quality control and filtering will be conducted with DADA2 (61) in the QIIME 2 platform (62). The amplicon sequence variants (ASV) will be aligned to construct a phylogenetic tree using fasttree from the QIIME 2 phylogeny plugin. Taxonomic assignment of ASVs will be performed using a Naive-Bayes classifier trained using the SILVA database (v. 138).

Differential expression analysis will be conducted with the negative binomial generalized linear models implemented in the DESeq2 package in R (63). Results will be adjusted for multiple testing using the false discovery rate (FDR) correction.

The association between Trp/5-HT/MLT/Kyn levels, expression levels of selected genes, inflammatory markers and clinical phenotypes will be analyzed with general linear models adjusting for age, sex and center of recruitment, by doing so we could reduce the bias arising from the wide age range chosen as inclusion criterion.

Multivariate differential abundance will be tested using Analysis of Composition of Microbiomes with Bias Correction (ANCOM-BC) and Linear Discriminant Analysis Effect Size (LEfSe).

For the prospective arm, repeated measures analyses such as Wilcoxon matched-pairs signed rank test will be used to assess longitudinal changes of the investigated markers.

### Ethics and dissemination

The studies involving humans were approved by the Ethical Committees of the San Raffaele Hospital (Prot SHERRIE GR-2019-12369523) and the University of Cagliari (Prot. PG/2021/14251). The studies will be conducted in accordance with the local legislation and institutional requirements. The participants have to provide their written informed consent to participate in this study.

To regulate the collection, conservation and use of human biological material and information on pathologies of interest and their course, the IRCCS San Raffaele Hospital has established a Biological Resources Center (BRC) Biological Bank (BB) aimed at the collection and conservation of human biological material used for diagnosis, biodiversity studies and research. Tailored databases containing the clinical and genetic information of patients whose biological material is stored at the BRC/BB have also been developed. Both the BRC/BB and the databases will be managed according to the current privacy protection regulations. Compliance with the rules set out in the relevant regulation is mandated for all applications submitted to the Ethics Committee of the San Raffaele Hospital when requesting the authorization to conserve biological material at the BRC/BB, in their own databases, or at both locations.

One purpose of the study, in accordance with good research practice, is to publish the results obtained in peer-reviewed international scientific journals, and to present these data at national or international conferences. We plan on engaging the service users taking part in our project and any interested stakeholders in disseminating the study results, along with their practical implications in terms of clinical outcomes and future biomarkers development in this area of research.

## Discussion

Despite the development of numerous antipsychotic drugs, significant challenges remain in treating key symptoms of SCZ, particularly cognitive dysfunction and metabolic syndrome, which are primary determinants of disability and reduced quality of life in affected individuals. Additionally, there is a notable lack of precision medicine approaches that can effectively tailor treatments to individual patient profiles. This deficiency is primarily due to the limited understanding of the neurobiological mechanisms underlying schizophrenia and its comorbidities.

Recent evidence indicates that there may be a common underlying mechanism involving the metabolism of tryptophan through the serotonin, melatonin, and kynurenine pathways, as well as the role of inflammation and its relationship with the gut microbiota (24, 28). Understanding these pathways and their potential link with SCZ and comorbidities could indicate new avenues for targeted therapies and precision medicine approaches.

This study seeks to explore these potential links from a translational perspective, aiming to bridge the gap between basic scientific research and clinical application. The present protocol details the clinical arm of our study, focusing on assessing these biochemical and microbial interactions and their impact on clinical outcomes in patients with SCZ. We must acknowledge certain limitations, especially concerning possible confounding factors that may affect the biomolecular data analyzed. The primary confounder is the wide age range of the participants, which will be however addressed in the analysis to account for age-related variations in inflammatory markers. Nevertheless, we expect to expand the evidence base for the potential worth as a candidate biomarker of TRP along the 5-HT/MLT and KYN pathways and inflammation in relation to clinically significant outcomes. Additionally, we will investigate the relationship between these biomarkers and gut microbiota composition, and how these interactions may influence distinct disease trajectories in clinical samples of individuals with SCZ. We hope that this protocol will be useful for researchers seeking to replicate the proposed study design proposed, thereby minimizing confounding variables deriving from different study methodologies. This would enable data collection under similar conditions using comparable techniques. Furthermore, we hope that these data will be relevant for researchers, clinicians, individuals with lived experience, and other stakeholders, helping to prioritize the most promising elements to explore in this field. Such prioritization is essential for developing more effective and personalized treatment strategies for managing the clinical aspects and pharmacotherapy of this complex disorder.
